# Understanding Factors that Shape Exposure to Zoonotic and Food-Borne Diseases Across Wild Meat Trade Chains

**DOI:** 10.1007/s10745-022-00361-1

**Published:** 2022-11-09

**Authors:** Nathalie van Vliet, Jonas Muhindo, Jonas Nyumu, Charis Enns, Francis Massé, Brock Bersaglio, Paolo Cerutti, Robert Nasi

**Affiliations:** 1 Center for International Forestry Research/International Center for Research in Agro Forestry/International Institute for Tropical Agriculture, Bogor, Indonesia; 2 Centre for International Forestry Research (DRC office), Bogor, Indonesia; 3 Global Development Institute, University of Manchester, Manchester, UK; 4 Geography and Environmental Sciences, Northumbria University, Newcastle, UK; 5 International Development Department, University of Birmingham, Birmingham, UK

**Keywords:** Wildlife trade, Wild meat, Supply chain, Zoonosis, Food safety, Democratic Republic of Congo

## Abstract

The rise of zoonotic disease-related public health crises has sparked calls for policy action, including calls to close wildlife markets. Yet, these calls often reflect limited understanding of where, precisely, exposure to risk occurs along wildlife and wild meat trade chains. They also threaten to negatively impact food security and livelihoods. From a public health perspective, it is important to understand the practices that shape food safety all along the trade chain, resulting in meat that is either safe to eat or managed as a potential vector of pathogens. This article uses ethnographic methods to examine the steps that lead a wild animal from the forest to the plate of an urban consumer in Yangambi and Kisangani in the Democratic Republic of Congo (DRC). Focusing on hunters, village-level consumers, transporters, market traders and urban consumers, we highlight specific practices that expose different actors involved in the trade chain to wild meat related health risks, including exposure to food borne illnesses from contaminated meat and zoonotic pathogens through direct contact with wild animals, and the local practices in place to reduce the same. We discuss interventions that could help prevent and mitigate zoonotic and food borne disease risks associated with wild meat trade chains.

## Introduction

Worldwide, wild meat use is an important interface for the successful transmission of zoonotic pathogens (Wolfe et al., 2004; Loh et al., 2015). At the level of wild meat users, transmission occurs through direct contact (skin-to-skin contact, scratches, animal bites, contact with body fluids) or through oral transmission with the unintended ingestion of pathogens present in water, hands, utensils or food in the absence of strict hygiene (Loh et al., 2015). Direct contamination may occur at different points along the wild meat trade chain, including at the time of harvest, exsanguination, dehairing, defeathering, evisceration and consumption (Bertran et al., 2017; Gao et al., 2016). The absence of regulated food safety standards along what are often informal trade chains during the harvesting, transportation, butchering and preservation of meat risks further exposing hunters, butchers, vendors and consumers to the transmission of zoonotic diseases and food borne illnesses (FAO et al., 2020). The variety of zoonotic pathogens that can be transmitted from wildlife depends on the reservoir species and includes not only highly contagious viral pathogens such as Ebola, monkeypox and SARS and related coronaviruses, but also bacteria (e.g. *Salmonella* spp.; *Bacillus. anthracis; Brucella spp.)* and parasites (e.g. *Echinococcus multilocularis; Trichinellosis spp.)* (Kruse et al., 2004) that are common causes of food-borne and gastrointestinal illnesses and diseases.

In sub-Saharan Africa, available studies examining zoonotic transmission and food contamination pathways related to wild meat use have mostly focused on quantifying exposure to certain reservoir species and broadly describing the types of contact with wildlife that may lead to zoonotic transmission. For example, in Nigeria, Friant et al. (2015) showed that butchering to sell meat, being injured, using body parts for traditional medicine, collecting carcasses found in forests and farms and keeping animals as pets were the most common forms of contact between people and wildlife. Similarly, in the Democratic Republic of Congo (DRC), Rimoin et al., (2017) found that eating wild meat, cooking it and butchering or skinning animals were among the most common ways people were in contact with wild animals. Based on the frequency of hunting or consumption, both studies found that community members were mostly exposed to rodents, duikers and non-human primates. In Ghana, bat hunting, selling and consumption are also widespread, with exposure to zoonotic pathogens experienced predominantly by women who do the butchering and men who are hunters (Kamins et al., 2015).

While the above-mentioned studies provide an understanding of the general patterns of exposure to zoonotic diseases at the level of hunters and their families, little is known regarding exposure at different levels of the wild meat trade chain. More specifically, there is limited understanding of how different actors along the wild meat trade chain – including those involved in butchering, handling, preserving, packing, storing and transporting wild meat – contribute to or detract from food safety for final consumers in urban areas. Recognising that hundreds of millions of people worldwide depend on wild meat for their food security, and their nutritional status is intrinsically linked to the consumption of safe food, understanding the diverse actors and practices that shape food safety risks and determine the safe preservation of wild meat is of grave importance.

In this article, we use ethnographic research methods and a trade chain perspective to examine the steps that lead a wild animal from the forest to the plate of urban consumers. We also describe the practices at all levels of the chain that play a key role in food preservation or contamination and that may shape exposure to zoonotic diseases. Our approach addresses the following questions: Does one’s role in the trade chain shape exposure to zoonotic pathogens and contaminated food? At which point along the trade chain is exposure to zoonotic diseases highest? What practices support food preservation and which ones may increase food contamination? Which local practices and infrastructural – or other – challenges contribute to reducing exposure to zoonotic diseases and ensuring food safety?

## Methods

### Study Site

**Fig. 1:**
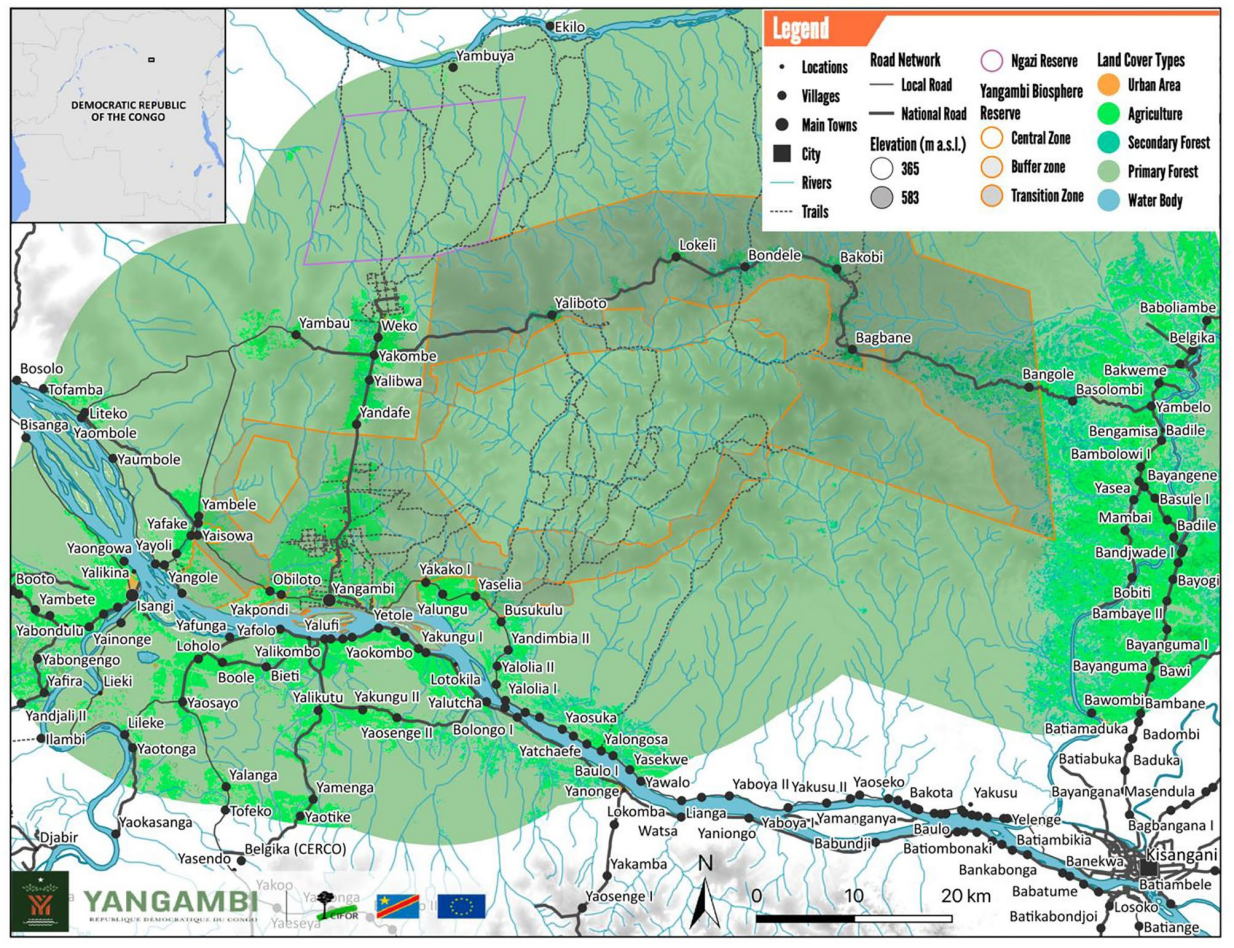
Map of Case Study Area (Produced by: CIFOR). Note: The limits of the reserve are for display purposes only. They are proposed limits that have not been officially approved by the Government

Our study focuses on the wild meat trade chain from Weko to Yangambi in DRC as described in van Vliet et al. (2019). The study area is located adjacent to the Yangambi Man and Biosphere Reserve, created in 1979. Yangambi is a town located in the northeast of the DRC, about 100 km West of Kisangani City in Tshopo Province (Fig. 1). The human population living around the Yangambi Man and Biosphere Reserve is estimated at 141 643 inhabitants, based on data from the Yangambi Registry Office dating from 2016. Yangambi was originally a research campus of INERA (Institut National d’Enseignement et Recherches Agronomiques) and IFA (Institut Facultaire de sciences Agronomiques) during colonial times, inhabited by staff and their families. Over time, the campus became a town as a result of the in-migration of workers and people searching for job opportunities. In the Yangambi landscape, roads and infrastructure are generally in poor condition, health establishments are insufficiently equipped, most households have no access to drinking water and the town of Yangambi is not electrified. The population in Yangambi is from mixed ethnic origins, but the rural population in villages surrounding the Reserve mainly identifies as Turumbo, regarded for their hunting skills. Traditional agriculture, including cultivating cassava, banana, maize, rice, cowpeas, beans and groundnuts, is the main activity in all villages around the reserve and contributes to household sustenance and livelihoods.

In the Yangambi landscape, two thirds of households experience insufficient food availability to meet 2,000 calories per day, falling below the recommended intake (Nowak et al., 2019). van Vliet et al. (2017) found that wild meat significantly contributed to the animal protein needs of the families with over 60% of households eating wild meat more than once a week. Wild meat demand from Yangambi Town is high because, apart from fish, there is barely any other source of animal protein available for the growing population. At the Yangambi market, fish costs almost twice as much as wild meat, with wild meat remaining the most affordable and accessible source of animal protein (van Vliet et al., 2017). As such, wild meat is consumed out of necessity in the whole Yangambi landscape, including Yangambi Town. To meet this demand, forests in the Yangambi landscape supply approximately 145 tonnes of smoked wild meat to consumers in Yangambi, the main consumption hub, per year (van Vliet et al., 2019). Most of the meat consumed in Yangambi originates from the Turumbo sector and particularly from Weko, a village located about 30 km north of Yangambi Town. As sales are very attractive in an area with few income earning prospects, hunters from Weko sell more than 80% of what they hunt, sometimes neglecting family food security needs (van Vliet et al., 2019). Until the early 2000s, wild meat from the Yangambi landscape travelled as far as Kisangani, just over 100 km away. However, due to increased local demand from Yangambi (and potentially shortfalls in supply), the amount of wild meat coming from Yangambi to Kisangani has decreased over the years.

As many as 34 species of wild animals are hunted and traded in local markets. The most traded species are small monkeys (*Cercopithecus ascianus*, *Cercopithecus neglectus*) (38% of the biomass), followed by red duikers (*Cephalophus* spp.), blue duikers (*Philantomba monticola*), bush pigs (*Potamochoerus porcus*) and bush tailed porcupines (*Atherurus africanus*) (van Vliet et al., 2019). Several species of small carnivores (*Crossarcus alexandri, Aonyx capensis, Genetta servalina, civectis civetta, Nandinia binotata*) and rodents (*Cricetomys emini*; *Thrynomys swinderianus*) are also commonly hunted. Chimpanzees, which are still present in the area although at very low densities, may also be hunted and traded. Frugivorous bats are also hunted, but only on a seasonal basis. Most meat is smoked and sold.

### Data Collection

Our methodology was inspired by Milstein et al. (2020), who used a mix of participant observation and semi-structured interviews to understand zoonotic transmission pathways at the hunters’ level in Guyana. Uniquely, however, our methodology took into consideration five different levels along the trade chain: hunters, consumers in villages, transporters, traders and consumers in urban areas. We mixed in-depth participant observation and semi-structured interviews at each of these levels, collecting data from February 2018 to October 2021. During this time, we (two of the authors of this paper) intermittently lived in Weko for about two years, learning about the context, culture and ways of life. Each time we visited Weko, we were hosted by the families of different hunters in the village. In total, we followed and observed 15 Turumbo hunters from Weko and participated in 9 distinct hunting trips. To avoid bias, we did not organize the hunting trips, but rather asked permission to come along on hunters’ routine hunting parties. This was done on an opportunistic basis, trying not to disturb the hunters’ activities and with prior consent from the chief of the village, the clan chief and the hunters. Most of the hunting trips lasted for about 3-4 days and consisted of staying at camps located between 10 to 25 kms from the nearest village. During the hunting trips, information was recorded about transportation practices between camp and village, butchering, smoking, meal preparation and consumption. Particular attention was paid to the handling of the carcasses, the places where they were stored, the tools used for their preparation, access to water and other cleansing strategies. During these hunting trips, we observed the butchering process for tortoise, small monkeys, blue duiker, giant and white bellied pangolins, brush tailed porcupines, turacos and red duiker. We also spent time with the hunters’ families at the village to observe the processing of the meat and the food preparation.

Considerable amount of time was also spent with the traders at the main markets in Yangambi and Kisangani (van Vliet et al., 2017; van Vliet et al., 2019). In particular, we had consistent interactions with 10 women in the Yangambi market and 15 traders at the main market in Kisangani. All traders were women. Spending time at their stalls regularly allowed us to observe the market dynamic, the state in which wild meat was exposed for sale, butchered, preserved and stored.

To complement our observations on practices at different levels of the trade chain, we prepared a list of guiding questions for semi-structured interviews with a sample of wild meat users at different levels of the wild meat trade chain. We used convenience sampling techniques described in Bernard (2011), including relevant stakeholders in the trade chain previously identified in van Vliet et al. (2019). In total, we interviewed 158 participants including hunters from Weko (n=15), their wives (n=15), market traders in Yangambi (n=10), wild meat cooks in Yangambi (n=3), final consumers in Yangambi (n=30), wild meat traders in Kisangani central market (n=15), wild meat cooks in Kisangani (n=5) and finally consumers in Kisangani (n=60). These interviews took place in October 2021 using the ®Kobotoolbox to design the interview guide, collect responses and store, share and export data for analysis. The interview included questions about socio-economic background and social position (e.g. ethnic groups, gender, main occupation, age), wild meat handling practices and food safety strategies. At the level of consumers in town (Yangambi and Kisangani), we asked people buying wild meat at the markets during our visits if they would agree to be interviewed at a time and place outside their homes convenient for them. The results of these questionnaires were analyzed to generate frequencies in practices and assess whether patterns observed through participant observation could be interpreted as relevant exceptions or as more generalized practices.

## Results

### Description of the Wild Meat Handling Process from the Hunter to the Final Consumer

#### From the Forest to the Village

**Fig. 2:**
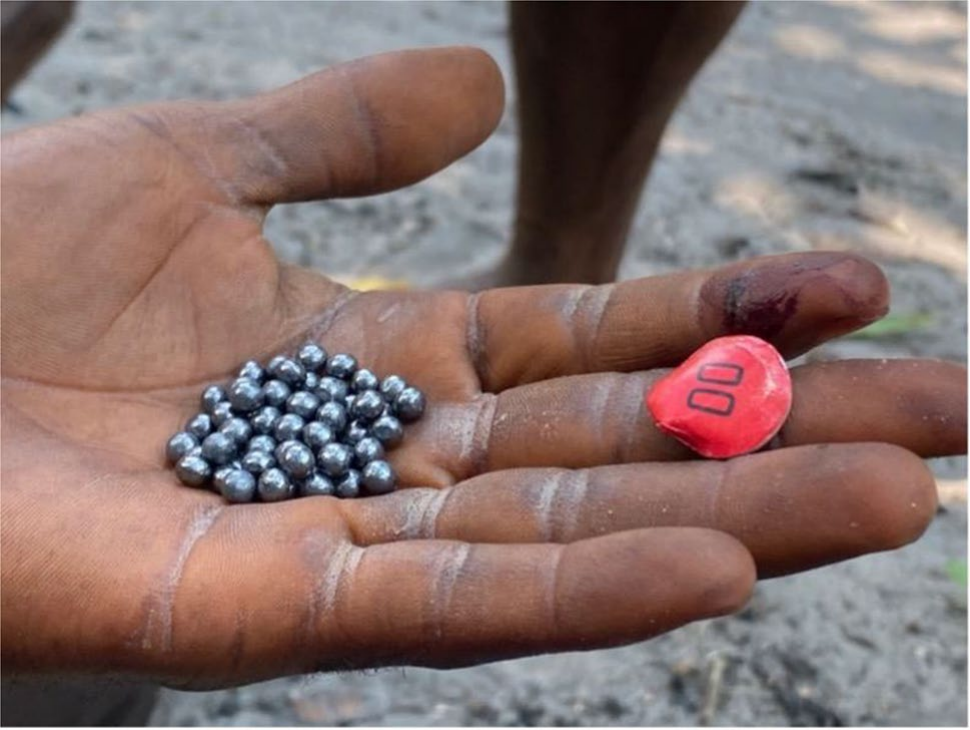
Double-0 Cartridges Commonly Used for Hunting (Photo credit: Nathalie van Vliet)

**Fig. 3:**
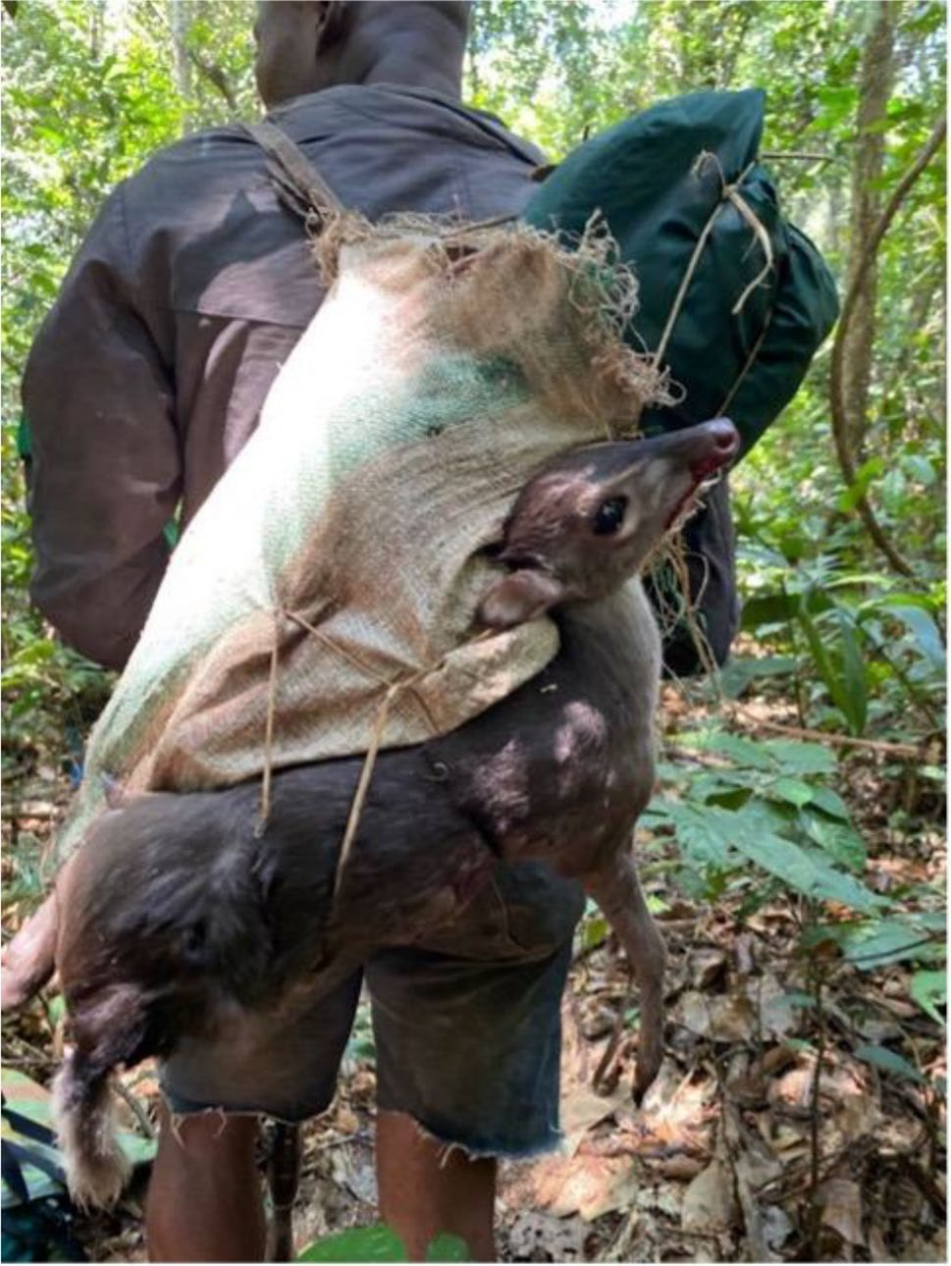
Hunter Carrying a Freshly Killed Blue Duiker (Photo credit: Nathalie van Vliet)

Hunters who participated in the wild meat trade during our research in the Yangambi region were all men between 25 and 40 years old. The use of guns is by far the most common hunting technique, but snares and dogs are also commonly used. When hunting with guns, game is shot either with purchased ammunition (Double-0) or locally manufactured ammunition (with pieces of recycled cartridges or any pieces of metal and matches used as explosive powder) (Fig. 2). Hunting usually takes place close to the hunting camps (usually less than five kilometers away) and the camps are located at about 10 to 25 kms from the village. Gun hunting usually takes place from dusk to dawn, but mostly at night with head lamps. When the animal is shot, it bleeds at different locations corresponding to the areas where it was hit by the different bullets. If the animal did not die immediately, the hunter kills it upon locating it. The dead animal, bleeding, is carried on the shoulder by the hunter to the hunting camp and left on the ground or hanged by a stick or branch ready for butchering (Fig. 3). If the animal is caught on a trap, the animal is usually not bleeding, but if found alive by the hunter, the hunter usually kills the animal with a machete. Time elapsed from when an animal is shot or caught in a trap to the time it is butchered varies between six to ten hours. This accounts for the transportation time to the camp and the fact that butchering usually occurs at mid-day when hunting is not productive, and hunters have spare time at the camp to start the butchering process. There was little variation of this process across hunters.

The process of butchering animals is done by the hunter or a porter (usually also a man between 18 to 25 years old) and takes place at the camp where there is access to water from a nearby stream. The carcass is placed over some large leaves on the ground. A bucket of water is placed near the butchering site. A very neat sagittal incision (about 10 cm) is made along ventral midline, starting just below the ventral to the mandibular symphysis and ending at the pubis, using a large butcher knife. The rib cage and pubis is split and the carcass is eviscerated with the hand making inside rips with the fingers inside the body wall to detach internal organs, which are set aside, usually placed in a bucket. The stomach and intestines are placed separately on the floor or in a separate bowl. For larger animals, the incision is not a line but rather an oval area that allows for all the internal organs to be removed. In rodents and monkeys, the gall is removed carefully and thrown away. In most hunted animals, all organs and parts are consumed, apart from hairs, scales, horns, hooves or spines. The head and front legs are chopped off, except for small animals (rodents, small carnivores and small monkeys for which the head and legs are kept together with the main carcass). For large animals (bush pig, sitatunga, etc.), the carcass is cut into six main pieces. During the butchering process, the butcher could cut himself with the knife or the bones inside the thoracic cavity. This could happen several times per month according to half of the hunters interviewed or several times a year according to the rest. No personal protection equipment (gloves, masks, apron) is worn during butchery.

While the main carcass is left on the floor lying over the large leaves, the internal organs are taken to the closest stream (usually a few meters from the camp). The heart, liver and lungs are thoroughly cleaned and kept in the bucket with water. The stomach is opened and its contents emptied into the stream. The stomach is folded inside out to wash all the walls carefully until no other visible contents are observed. All tools are washed with running water in the stream. The internal organs are cut into pieces and boiled in salty water for consumption at the camp by the hunter and porters. The head, neck and legs are also usually roasted at the camp to burn all hairs and boiled together with the internal organs. The hunter keeps a few pieces of these organs to bring home and share with their family. In this case, the cooked pieces are packaged in leaves for transportation. When dogs participate in hunts, they are fed with the same cooked food that hunters consume and no raw meat is given to them.

**Fig. 4:**
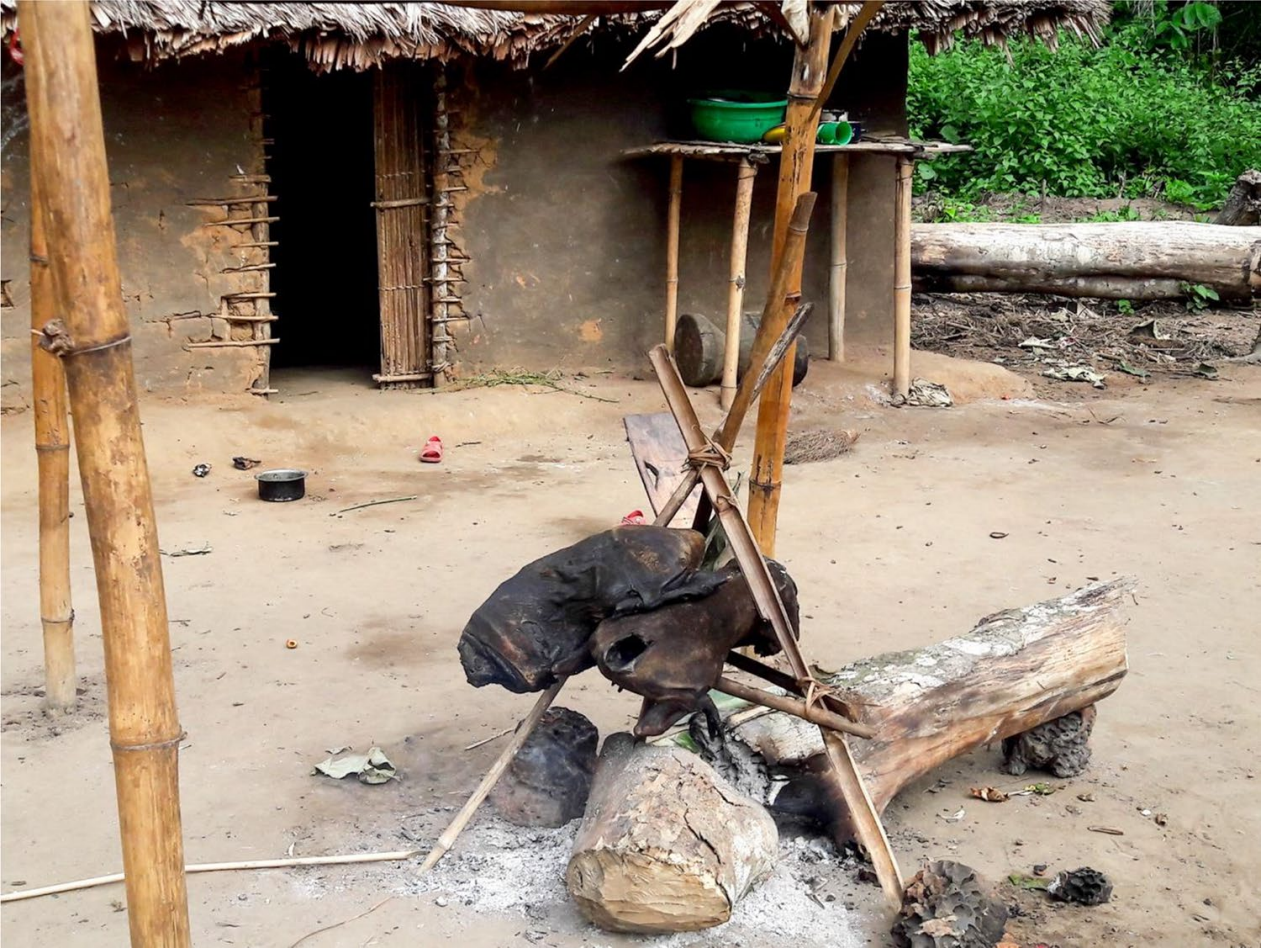
Carcasses After Smoking (Photo credit: Jonas Nyumu)

The main carcass (or the pieces of carcass for large animals) is roasted to remove all hairs. At the camp, the carcasses are smoked for as long as the hunting trip takes place (usually for about two days) (Fig. 4). If the carcass is butchered on the last day of the hunting trip, then it is only roasted for a few minutes, allowing hair to be removed but keeping the meat raw, as traders usually prefer raw meat. The smoking methods consist of placing four Y-shaped sticks vertically, then two sticks horizontally across them in the notches of the Y sticks. The carcass is placed over this structure at about one meter high over the fire. Although careful attention is given to butchering and smoking processes, none of the hunters expressed concern for the preservation of the meat. According to the hunters, smoking for two days allows the meat to be preserved for about seven to ten days. Carcasses are usually covered with leaves or put inside a bag to avoid contact with flies that lay their eggs in the meat. They are transported to the village inside those bags and in traditional baskets. Between one to four days may elapse (hunting trips last from three to five days) until the smoked or roasted carcasses reach the village. On rare occasions, the carcass is fresh, and this is only the case for animals shot the night before returning to the village. Most of the meat is smoked for a maximum of two days.

#### At the Hunter’s Household Level

At the hunters’ household level, the family consumes what the hunter has not sold, mostly small sized animals, such as African pouched rat or blue duiker, as well as certain parts of the animals that are not for sale, such as cooked viscera, head and legs from different game species. In some households, the children participate in complementing access to animal protein by hunting or fishing. Their hunting methods include nylon traps, slingshots and dogs. They hunt small animals such as birds, squirrels, African pouched rats and aulacodes, that they prepare themselves or give to the women in charge of cooking in the household. Families in the villages also get animal protein from seasonal wild meat species such as frugivorous bats that are killed with slingshots. All family members participate in these bat hunts.

The hunter’s wife is usually in charge of the preparation of meals at home. Sometimes meal preparation is done by the hunter’s sister or mother instead. For preparation, the meat is butchered in the kitchen, being cut with a knife or machete. Among the women interviewed (n=14), 13 prepared wild meat at least once a week and only one mentioned preparing it once or twice per month. When wild meat is brought back by the hunter to the family, the meat is already smoked in most cases. However, a few women mentioned that they would sometimes also get freshly killed wild meat. This fresh meat is in principle prepared the following day after arrival but could be kept for the subsequent day. The carcass is covered with leaves and put in a dry area close to the fire. Keeping the meat in food safe conditions was a concern for most women we interviewed. They were worried that its taste would change, that the meal would be spoiled after so much effort or that the family would get sick if they still ate it. A few women mentioned that they would still prepare the meat even if it had started the decomposition process: they would smoke it again before preparation. They mentioned that having to get rid of meat that is decomposed and useless for consumption only happens less than three times a year. Signs of decomposition include the presence of maggots, a very strong smell and a change in the consistency of the meat, which they described as ‘fluffy’.

Domestic animals such as dogs and chicken are usually fed with the remains. The pieces of meat are directly placed in the pot or on leaves. Everything is consumed, except the horns, spines, hooves, nostrils and reproductive organs of the animal. When rodents and monkeys are fresh, the gall is removed carefully and thrown away. After butchering, the knife and hands are washed with water (soap was also used by most women interviewed). The pieces of meat are cleaned with water before cooking and then boiled for several minutes in salty water. There is no running water in the village. Water is fetched from nearby streams, and it takes women more than one hour to fetch water and return home. After boiling the meat, the water is removed and stir fried with other forest condiments and then prepared either with legumes (manioc leaves), peanut or pumpkin seeds. The meal is served a couple of hours after preparation, but not all is consumed the same day. The leftovers are kept on the pot and heated before consumption the next morning.

#### From the Village to the Market

Wild meat is transported from the village to Yangambi (30 km) on motorbike or bicycle once a week. Transporters are Lokele or Turumbo men coming from Yangambi to Weko loaded with all sorts of manufactured goods needed in the village and then returning to Yangambi with a full load of wild meat. The road is in very bad condition and requires traveling in muddy or dusty conditions under the sun or rain. Some traders walk to Weko and hire a transporter back to Yangambi to reduce transportation costs. The age of the transporters that we interviewed varied between 36 to 57 years old. On average, they could transport about 50 kg of meat per trip. The species transported mainly include duikers, African pouched rat, brush tailed porcupine, small monkeys and bush pigs.

**Fig. 5:**
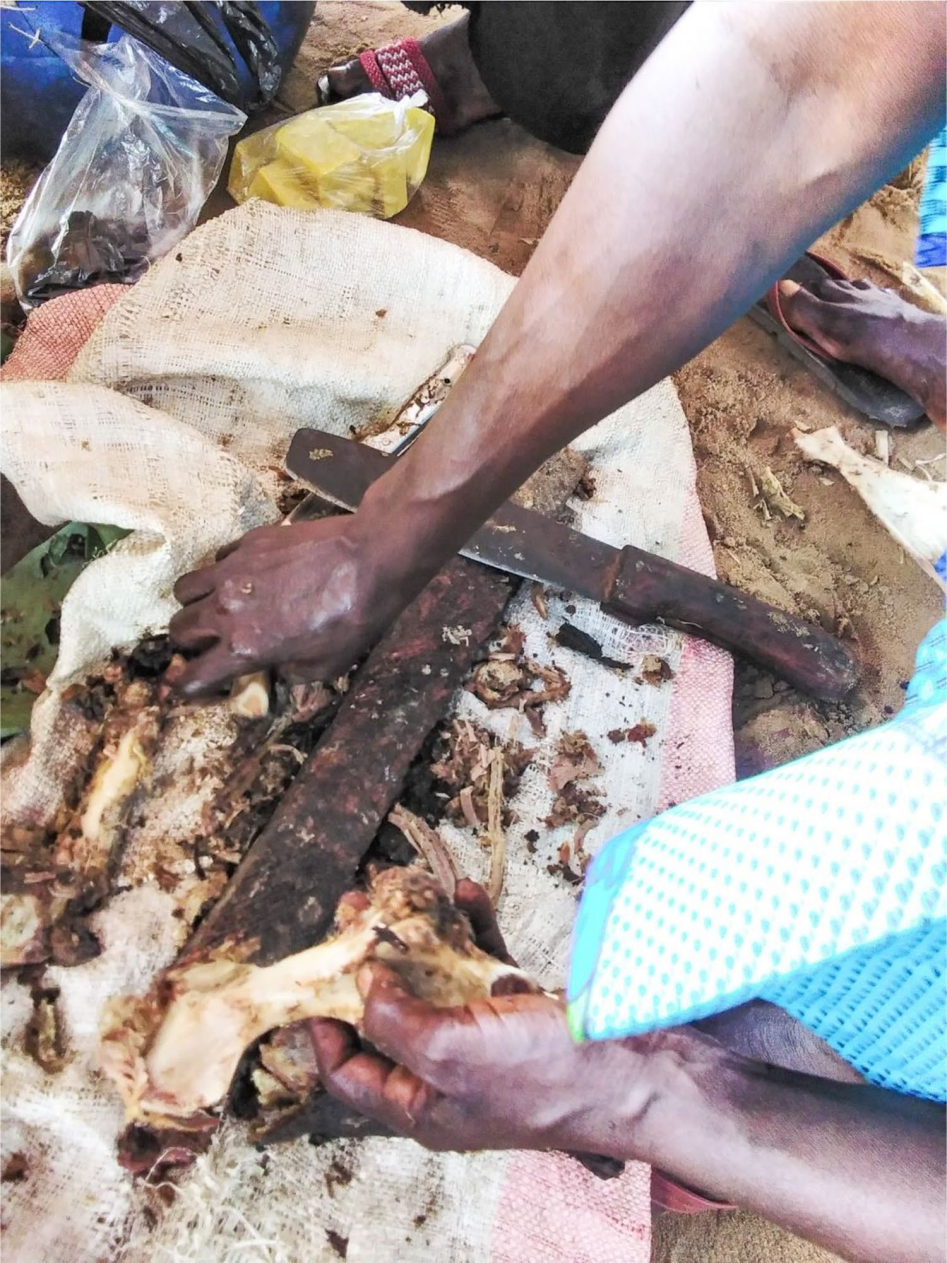
Smoked Carcasses Butchered at Market (Photo credit: Jonas Nyumu)

Ensuring that the meat does not spoil was a concern for half of the transporters interviewed. Some of the strategies mentioned to avoid decomposition include smoking the meat again before transportation and stocking it close to the fire. Most of the meat they transport is already smoked and careful attention is given to packaging meats in different stages of preservation separately. All smoked carcasses are placed in the same basket or bag, although without consideration for separating different species (Fig. 5). A mosquito net is sometimes used to avoid flies. If available, roasted meat is put at the bottom of the baskets or bags and separated with large leaves from the smoked meat, which is placed at the top. When fresh animals are available, these are not mixed with the other carcasses. Instead, they usually hang separately on the bike or motorbike on which the wild meat is transported. If transported on a motorbike, the meat reaches Yangambi in two hours, but on bicycle it could take one or two days, depending on the load. The wild meat is delivered to the wild meat traders who sell it at different markets around Yangambi.

#### At the Market

Wild meat traders in Yangambi are from the Lokele, Topoke Mumbole and Musoko ethnic groups. The age of those interviewed varies from 29 to 45 years old. Most sell wild meat several times a week. Wild meat comes from Weko, but also from other source areas such as Bengalema. Most of the meat they sell is smoked. However, when the meat comes from Weko they could have a chance to have fresh meat for sale. The meat purchased by traders is usually sold to the final consumer in about one or two days. To preserve the meat, traders choose dry areas close to the fire to store the meat. In a few cases, mosquito nets are used to protect the meat from flies and two of the traders have a generator which allows them to freeze the meat. Market stalls are simple wood tables, protected from the sun and rain by umbrellas. Running water is not available at the market, but the Congo River flows about 10 to 50 meters from the market. The majority of traders mentioned that they are obliged to get rid of meat that decomposes before sale at least once per year. Wild meat is butchered at the market stalls. Traders interviewed reported being cut with their knives two to three times per year. Dogs, chicken, mice and ducks feed on any wild meat remains. Two out of 14 traders reported not washing their hands or tools with water after butchering and only a minority wash them with water and soap. All parts of the animals are sold, except the big bone, which is thrown on the fire, and the sternum, which is consumed by their own family. For most traders, food safety aspects are a concern because they could lose the meat and subsequent income, and if the meat is further smoked, it would lose volume and value.

#### Consumers in Town

Consumers in Yangambi are women from the Topoke, Lokele, Mukongo, Budu and Nande ethnic groups. Those that we interviewed (n=34) ranged from 27 to 58 years old. The majority prepare smoked wild meat more than once per month. Only a few women interviewed get fresh wild meat. The meat is preserved by covering it with leaves in a dry area close to the fire. Most of the consumers are worried about the food safety conditions surrounding preservation of the meat. They mentioned the need to throw out the meat when it has already started to decompose as the meat loses its flavor and could cause diseases leading to diarrhea or vomiting. All women interviewed mentioned that they get rid of decomposing meat at least a few times per year. Consumers in Yangambi use the same indicators of decomposition as traders: presence of maggots, a very strong smell and a change in the consistency of the meat which becomes fluffy. The butchering process is similar to that observed in Weko. During the butchering, most women interviewed mentioned getting injured a couple of times per year with their knives while cutting the meat. Here also, chicken and dogs feed on the remains. Tools and hands are cleaned with water and soap. All parts of the animals are consumed, except the horns, scales, spines and gall (for rodents and monkeys). The big bone is also often removed. These parts are thrown anywhere around the kitchen or in the fire. The smoked flesh can be consumed as such without further cooking. The cooking practices are similar to those observed in the village. Access to running water varies with each household from readily available nearby to having to fetch it as far as one hour away.

In Kisangani, wild meat handling practices are similar to those observed in Yangambi. However, there are a few key differences. In Kisangani, most women purchase smoked wild meat, whereas fresh meat is more common in Yangambi. Additionally, the majority are worried about food safety, and all have soap to wash their hands and tools. Tapped water is available in the house or at the water pump (less than 10 minutes from the house). Two women said they could preserve meat in a freezer.

### Overview of Handling, Preserving, Transporting And Storing Practices and Exposure to Health Risks

We complemented our observational research with semi-structured interviews with stakeholders to understand the handling, preserving, transporting and storing practice of wild meat along the trade chain. We also discussed the various experiences with and perceptions of exposure to wild meat related health risks at each level of activity and how these are managed. This analysis provides insight into where along the wild meat chain risks of zoonotic disease and food borne illness is highest, who is likely to be most affected, the practices in place that might help reduce or increase this risk and opportunities for further intervention.

The frequency of contact with wild meat and bodily fluids was highest at the level of hunters and market traders, and hunters were much more exposed to fresh meat (Table 1). During the butchering process, hunters (all men) were the most exposed to wounds, particularly when they inserted their hand or knives in the thoracic cage of the animals to remove the guts of fresh carcasses. Intestinal worms were the most common pathogen observed in wild meat during the butchering process, particularly in the intestines of chimpanzees, baboons and other small monkeys. Maggots and cysts were observed in the flesh. Magots could also be observed in the brain, heart and intestines, particularly in bush pigs. Cartridge balls were also found in the flesh. Some animals had gall on their skin or could carry ticks. Exposure to fresh meat decreased along the trade chain with consumers in urban areas rarely being exposed to body fluids from wildlife. Potential transmission of zoonotic diseases from direct contact is thus highest at level of the hunters (men) and their porters as compared to other users, because most of the butchering of fresh animals is done at their level and at the hunting camps.

**Table 1:** Exposure and Prevention Along the Wild Meat Trade Chain

	**Hunters**	**Village consumers**	**Transporters**	**Traders in Yangambi**	**Consumers in Yangambi**	**Consumers in Kisangani**
**Frequency of wild meat handling (**the category with highest percentage of respondents is shown here)	Almost every day (100%)	At least once a week (100%)	Once a week (100%)	Almost every day (100%)	At least once a week (27%) At least once a month (46%) Every 2-3 months (27%)	At least once a week (10%) At least once a month (55%) Every 2-3 months (30%) Once a year (5%)
**% of fresh meat at each level of the trade chain**	100%	35%	30%	20%	20%	5%
**Frequency of wounds while butchering**	Several times a month (15%) 2-3 times a year (50%)	2-3 times a year (100%)	NA	2-3 times a year (100%)	Several times a month (9%) 2-3 times a year (91%)	2-3 times a year (100%)
**% of users concerned by the food safety in wild meat**	0%	24%	50%	78%	90%	91%
**List of strategies to avoid food contamination**	Washing the food thoroughly before cooking Removing gall (rodents and monkeys) and hair Placing the meat on leaves while butchering Boiling the meat Smoking the meat Covering the smoked carcass from flies	Washing the food thoroughly before cooking Removing gall (rodents and monkeys) and hair Placing the meat on leaves or pot while butchering Boiling the meat Smoking the meat Covering the smoked carcass from flies and placing it close to fire Leaving the cooked food in covered pot close to fire Heating the food again before consumption	Smoking the meat Packaging the meat in bags, leaves, mosquito nets Not mixing fresh meat with smoked meat Separating roasted meat from smoked meat	Smoking the meat Covering the smoked carcass from flies and placing it close to fire	Washing the food thoroughly before cooking Placing the meat on the pot while butchering Boiling the meat Smoking the meat Covering the smoked carcass from flies and placing it close to fire Leaving the cooked food in covered pot close to fire Heating the food again before consumption	Washing the food thoroughly before cooking Placing the meat on the pot while butchering Boiling the meat Smoking the meat Covering the smoked carcass from flies and placing it close to fire Leaving the cooked food in covered pot close to fire (but some may leave it in their freezer) Heating the food again before consumption
**Tools cleaned (use of water and soap (% of persons mentioning use)**	With abundant water (100%)	With water (100%) and soap (77%)	NA	With water (86%) and soap (28%) Do not clean their tools (14%)	With water (100%) and soap (88%)	With water (100%) and soap (100%)
**Access to running water measure in terms of time needed to reach the nearest water source (min)**	1 min (streams in the forest)	60 min	NA	No water in market stall 5 min (Congo river)	1 (well) to 60 min (streams)	10 min (tapped or pump)
**Access to electricity**	0%	0%	14% (generator)	0%	0%	10% (electricity)

Despite the risks of direct contact with bodily fluids at the level of hunters and their camps, no hunters or traders expressed concern about the possibility for zoonotic diseases to spill over from wildlife to humans through wild meat. Instead, all actors placed particular attention and interest in ensuring that the meat was well preserved, both to reduce economic loss in case of spoilage and to prevent health consequences of eating contaminated or rotten meat. From the forest to the final consumer, wild meat was smoked several times at each level of the chain: 4 to 6 times, equivalent to more than 72h of smoking. The process of smoking lasted at least two days at the level of the hunters, but the meat was systematically smoked again for several hours and placed close to the fire before it reached the final consumer. A carcass killed in the Weko forest reached the final consumer in Yangambi between 8 to 12 days after it was killed and the final consumer in Kisangani in 13 to 17 days.

In contrast to the low percentage of hunters concerned with zoonoses and food safety risks, traders (78%) and end consumers of wild meat in both Yangambi (90%) and Kisangani (91%) expressed concern, and this was largely about food safety and food borne illnesses related to meat preservation and food contamination. Several practices were put in place by stakeholders at all levels of the chain to ensure food safety conditions, but these practices may also prevent the transmission of zoonotic diseases. Hunters carefully removed the anus and intestine to reduce fecal contamination with enteric pathogens. The hide and skin were kept on the carcass, protecting the meat against contamination. At the level of the transporters, raw meat was not mixed with smoked or roasted meat to prevent contamination of other meat with drops of bodily fluid. At the level of consumers, the meat was kept in a dry area, covered with leaves or mosquito nets to avoid flies and the meat was cleaned with water and then boiled at high temperature to reduce the superficial bacterial load. In addition, most of the meat was smoked several times before consumption, which improved the preservation of the meat during transportation.

Access to electricity is a key limiting factor to reduce food contamination and only available in Kisangani at the end of the trade chain. Water access is also a factor. While hunters had access to running water from streams near hunting camps, women in charge of food preparation at the village level and in Yangambi usually needed to fetch water from remote sources. In Kisangani, all households interviewed had access to tapped water or from a nearby pump. However, market traders did not have access to clean water, and this prevented them from washing tools and hands during the butchering of the meat, thus increasing risks of contaminating meat.

## Discussion

Recognising the importance of understanding how people of different ages, genders, socio-economic position and livelihood are differentially exposed to environmental hazards and risks (Robbins, 2019; Sultana, 2021) – and specifically to risks of zoonotic disease transmission (Dzingirai et al., 2017a, 2017b; Leach et al., 2017) – our analysis contributes to understanding of the different levels and dynamics of wild meat risk and exposure to zoonotic diseases and foodborne illnesses, including where along the wild meat trade chain these occur, during what activities and who is most exposed and/or vulnerable. Understanding where different risks of exposure occur, who is most impacted and through what practices allows for the development of effective interventions to reduce public health risks along the wild meat trade chain.

In this study, we identified the segments of the population that are most exposed to food safety risks, including exposure to zoonotic pathogens transmitted through direct contact, along with activities along the chain that increase exposure. Our results illustrate the various steps and practices in which food contamination can occur and how different stakeholders are affected based on the activity one is involved in. As we show, exposure varies by one’s gender, age, role in the trade chain and access to utilities and infrastructure, such as water and electricity. Furthermore, paralleling insights from Leach et al. (2017), we show that risks change according to the temporality and spatiality of activities, including the duration of different activities that one is involved in and the spaces where these activities take place. For example, the risk of zoonotic disease transmission is highest at the level of the hunters (men) and their porters as compared to other users, because most of the butchering of fresh animals is done at their level and at the hunting camps. This differs from findings in Guyana, where Milstein et al. (2020) demonstrate that wild meat is butchered in the village mostly by women. Given that guts from dead animals need to be removed in the 20 hours following death (Milstein et al., 2020), the distance of hunting camps to the village and the time needed to procure enough meat to satisfy the hunter’s need likely determine the place where the carcass is butchered and who is involved in this process. In our study, hunting camps were located about 25 km away from the village and hunting trips last between three to five days, meaning that the butchering process needs to take place at the hunting camp with associated risks of that context. Risks of foodborne illness on the other hand is highest among final consumers, potentially affecting the entire household.

The analysis in this article also captures specific strategies implemented by those involved across the wild meat trade chain to prepare and preserve meat and increase food safety, along with practices that may contribute to the opposite. For example, the smoking process clearly represents the best available preservation method. Still, more research is needed to understand the influence of wood type and length of the smoking process on the textual, sensory, nutritional, antioxidative and antimicrobial properties of the smoked food (Maga, 2018). Similarly, a preference among traders for fresh or raw meat must be recognized as a pressure that hunters face when deciding whether or not to leave meat unsmoked, which similarly has implications for zoonotic spillover and food safety.

The butchering process provides a further example of activities that takes place along the supply chain that can contribute to or detract from food safety for final consumers in urban areas. As observed in other contexts, the butchering process in the wild meat chain in Yangambi follows a careful protocol which aims to ensure that the meat consumed is safe and can be preserved over the whole duration of its journey from the forest to the plate of the final consumer (Paulsen et al., 2012; Carrasco-Garcia et al., 2018; Hedman et al., 2020; a Mpalang et al., 2013; Chaber & Cunningham, 2016). As described above, this includes measures like only feeding cooked scraps to dogs to avoid transmission of pathogens among domestic animals. These practices that hunters put in place to ensure the safe preservation of the meat may also serve to prevent or increase spillovers risks from wildlife to humans. As such, the careful analysis of such practices can serve as starting points to develop culturally appropriate and locally relevant strategies to reduce the risk of spill overs.

Our analysis also highlights infrastructural challenges that contribute to unnecessary exposure to zoonotic and food-borne illnesses, revealing clear areas of intervention for donors and public health agencies. Access to electricity, for example, is a key limiting factor to reduce food contamination and is only available in Kisangani at the end of the trade chain. Water access is also an issue. While hunters have access to running water from streams near hunting camps, women in charge of food preparation at the village level and in Yangambi have limited access to water and must travel to water sources. In Kisangani, all households interviewed had access to tapped water or from a nearby pump. Market traders on the other hand, do not have access to running or clean water, detracting from food hygiene as they are less able to effectively wash tools and hands during the butchering of the meat. These infrastructural issues, including a lack of access to running water and electricity, represent a major barrier to food safe butchery; for example, making it difficult to safely dispose of animal parts that could result in the transmission of pathogens to domestic animals, scavengers and other wildlife in the landscape (Paulsen et al., 2011). The lack of electricity and running water in markets also contributes to higher than necessary risks of food borne illnesses among final consumers of wild meat.

In the absence of formal food safety regulation, the development of guidelines for self-inspection based on local and scientific knowledge is an area that may require further research (Winkelmayer et al., 2011). Careful analysis of practices along the food chain can serve as a starting point to develop culturally appropriate and locally relevant strategies to reduce risks for all involved in the trade chain, including the end consumer. For instance, where access to water is an issue, rubber gloves and use of dedicated knives for wild meat butchery and preparation should be encouraged to avoid cross contamination (Hedman et al., 2020). This type of basic, practical intervention, in addition to provision of water and electricity, can be supported by relevant agencies and donors to reduce the public health risks of wild meat and improve food safety, including reducing the risk of contamination and zoonotic disease transmission.

Another important finding of our study is that wild meat users (consumers in particular) tend to be more concerned about food borne diseases in wild meat rather than the transmission of zoonotic pathogens. While ‘spectacular’ zoonotic disease outbreaks, such as Ebola, may grab headlines, wild meat users are mostly wary of food-borne gastrointestinal illnesses that may be caused by unsafe food-handling practices. Compared to the risks from butchering, the risk for food contamination and the oral transmission of zoonotic diseases increases at the level of consumers. Poor food preservation and food contamination is of high concern for the final *consumers*, particularly in towns due to the risk of decomposition which may lead to food loss and to health consequences at the household level. Exposure of the meat to rain, dust, flies and cross contamination with other foods is highest during the transportation and at market stalls. Fecal contamination of wild meat is also often above recommended levels in many sub-Sahelian markets (van Vliet et al., 2017). For example, in Lubumbashi, a Mpalang et al. (2013) confirmed high levels of contamination of smoked wild meat carcasses by *Escherichia coli, Salmonella* spp., *Campylobacter jejuni* and *Campylobacter coli*.

Nevertheless, our results show that strategies to reduce zoonotic spillover are related to strategies to improve food preservation and food safety conditions. Because the latter is of primary concern to wild meat users, it represents a good entry point to engage stakeholders in a constructive transformation of the sector for the benefit of human health and local livelihoods. This finding also invites further efforts to understand and address the issues along trade chains that increase food born disease risks at the level of consumers. From an economic perspective, addressing infrastructural factors that determine food preservation is of paramount importance for those involved in wild meat trade as a safety net in the DRC and similar contexts.

## Conclusion

Utilising ethnographic methods and a trade chain perspective, we offer a comprehensive description of wild meat handling along an entire trade chain in DRC – from the forest to the final consumer. This methodology was adapted to reduce intrusiveness and increase the level of trust needed to generate detailed information at each point of the trade chain, while still effectively gathering the needed data on wild meat handling practices at all levels in the trade chain. We combined this with semi-structured interviews to better understand and quantify certain aspects of wild meat handling, food safety and exposure to zoonotic and food borne illness risk along the trade chain. Our analysis provides a thorough understanding of the ‘who’, ‘where’, ‘when’ and ‘how’ of exposure to zoonotic and food-related health risks along the wild meat value chain as well as the practices that can increase or decrease such risks. We demonstrate how ethnographic approaches may help tailor public health interventions aimed at increasing food safety to the local context, building on existing practices while providing culturally appropriate recommendations to increase food safety along the wild meat trade chains, including reducing zoonotic disease risk.

## Data Availability

The data that support the findings of this study are available from the corresponding author upon request.
